# Comparative study of 360° virtual reality and traditional two-dimensional video in nonface-to-face dental radiology classes: focusing on learning satisfaction and self-efficacy

**DOI:** 10.1186/s12909-023-04851-8

**Published:** 2023-11-12

**Authors:** Ji-Eun Im, Ja-Young Gu, Jung-Hee Bae, Jae-Gi Lee

**Affiliations:** 1 Department of Dental Hygiene, Graduate School of Namseoul University, Cheonan, Republic of Korea; 2 Department of Dental Hygiene, Sahmyook Health University, Seoul, Republic of Korea; 3 Department of Dental Hygiene, College of Health and Health Care, Namseoul University, Cheonan, Republic of Korea

**Keywords:** Dental radiology, Head-mounted display, Learning satisfaction, Self-efficacy, Smartphone, Virtual reality

## Abstract

**Background:**

Acquiring adequate theoretical knowledge in the field of dental radiography (DR) is essential for establishing a good foundation at the prepractical stage. Currently, nonface-to-face DR education predominantly relies on two-dimensional (2D) videos, highlighting the need for developing educational resources that address the inherent limitations of this method. We developed a virtual reality (VR) learning medium using 360° video with a prefabricated head-mounted display (pHMD) for nonface-to-face DR learning and compared it with a 2D video medium.

**Methods:**

Forty-four participants were randomly assigned to a control group (n = 23; 2D video) and an experimental group (n = 21; 360° VR). DR was re-enacted by the operator and recorded using 360° video. A survey was performed to assess learning satisfaction and self-efficacy. The nonparametric statistical tests comparing the groups were conducted using SPSS statistical analysis software.

**Results:**

Learners in the experimental group could experience VR for DR by attaching their smartphones to the pHMD. The 360° VR video with pHMD provided a step-by-step guide for DR learning from the point of view of an operator as VR. Learning satisfaction and self-efficacy were statistically significantly higher in the experimental group than the control group (p < 0.001).

**Conclusions:**

The 360° VR videos were associated with greater learning satisfaction and self-efficacy than conventional 2D videos. However, these findings do not necessarily substantiate the educational effects of this medium, but instead suggest that it may be considered a suitable alternative for DR education in a nonface-to-face environment. However, further examination of the extent of DR knowledge gained in a nonface-to-face setting is warranted. Future research should aim to develop simulation tools based on 3D objects and also explore additional uses of 360° VR videos as prepractical learning mediums.

## Background

Oral radiographs offer valuable diagnostic insights into dental diseases and conditions [1], often enabling the detection of oral diseases that are invisible to the naked eye [2]. Inaccurate oral radiographic images that fail to identify oral cavity structures necessitate rescanning, resulting in increased radiation exposure to the patient [3]. Hence, the dental radiology curriculum includes the fundamental principles of physics, equipment operation, radiation safety, dental radiography (DR) procedure, and radiology to facilitate accurate oral radiographic image recording [4].

The coronavirus disease 2019 (COVID-19) pandemic has made it difficult to conduct face-to-face classes, and digital education is compulsory [5]. The sudden transition to a nonface-to-face environment compelled instructors to utilize the conventional two-dimensional (2D) video medium or videoconferencing for DR education. However, despite the conclusion of the COVID-19 pandemic, the constraints associated with conventional 2D videos including reduced media richness and diminished immersion in the learning process persist [6]. Media richness refers to the inherent ability of digital media to simultaneously convey audiovisual cues and information, facilitating active participation and comprehension [7]. Specifically, 360° videos, classified as rich media, evoke diverse emotional responses from users by offering more extensive information and visual effects compared with conventional 2D videos [8]. These 360° videos are designed to enable users to engage in a virtual reality (VR) experience.

There is a demand for a digital education strategy that uses VR technology to facilitate 3D learning in the preclinical stage and adequately addresses all environmental constraints [9–12]. VR-based education can be used without spatial restrictions [13]. Users can experience a 3D immersive experience using visual data provided in three dimensions while wearing a head-mounted display (HMD) [14]. It is possible to create a learning environment that closely resembles real-world situations, thus increasing the opportunity for context-based learning and the transfer of skills and knowledge [15]. 360° VR videos are more realistic than 3D objects because they record the surrounding environment with a 360° camera, and its production process is simpler than that of VR using 3D objects [16]. However, in contrast to 3D object–oriented VR technology, the interactive functionality is constrained. 360° VR video can be easily accessed through the YouTube application on mobile devices and HMDs [17]. Of the various commercially available HMDs, prefabricated HMDs (pHMD) represent the most cost effective, easily manufacturable option, ensuring convenient access for students [18]. Rupp et al., reported that 360° videos were more immersive than 2D videos as they allowed the exploration of the different parts of a particular scene; however, low-cost pHMDs are somewhat lacking in terms of immersive VR learning experiences when compared to the more expensive HMDs [19]. However, within the context of this research, considering the convenience of development for instructors, affordability of pHMD, and ease of access for learners using smartphones, we aimed to develop a new learning medium that enables VR-based learning in a nonface-to-face online environment.

Online learning in a nonface-to-face environment necessitates the utilization of internet-based technology, thus requiring a certain degree of self-efficacy [20]. Although this may not always accurately predict final grades and satisfaction [21, 22], it exhibits a positive correlation with online learning performance [23, 24]. Notably, individuals who exhibit higher levels of satisfaction are more inclined to persist in their learning endeavors, suggesting that enhancing satisfaction levels can potentially diminish dropouts during the learning process [25]. In nonface-to-face online learning, the learning satisfaction and self-efficacy of students are important factors, with high levels facilitating continuous learning in an online environment [26]. Learning satisfaction is the degree until which learning environment, motivation, and achievement can affect academic achievement [27]. Self-efficacy is a cognitive trait related to persistence and motivation to overcome challenges, and it represents confidence to achieve goals [28]. In the learning process, learner satisfaction is an important learning goal, and learning style, self-efficacy, and active learning affect satisfaction [29]. In addition, the expectations and value beliefs of learners, including course value, course expectations, achievement expectations, cost expectations, and self-efficacy, play a mediating role between academic achievement and satisfaction [30]. Furthermore, self-efficacy influences motivation. Satisfaction with learning can also accurately capture student feedback, provide a framework for the educational process, and enhance class completion rates [31]. Evaluating individuals based on their self-efficacy and learning satisfaction holds more diverse pedagogical implications than assessing them solely based on scores that reflect academic achievement.

This study aimed to evaluate the potential utility of 360° videos using pHMD for DR education by comparing them with 2D videos. The outcome measures of interest were self-reported learner satisfaction and self-efficacy.

## Methods

### Research design

**Fig. 1:**
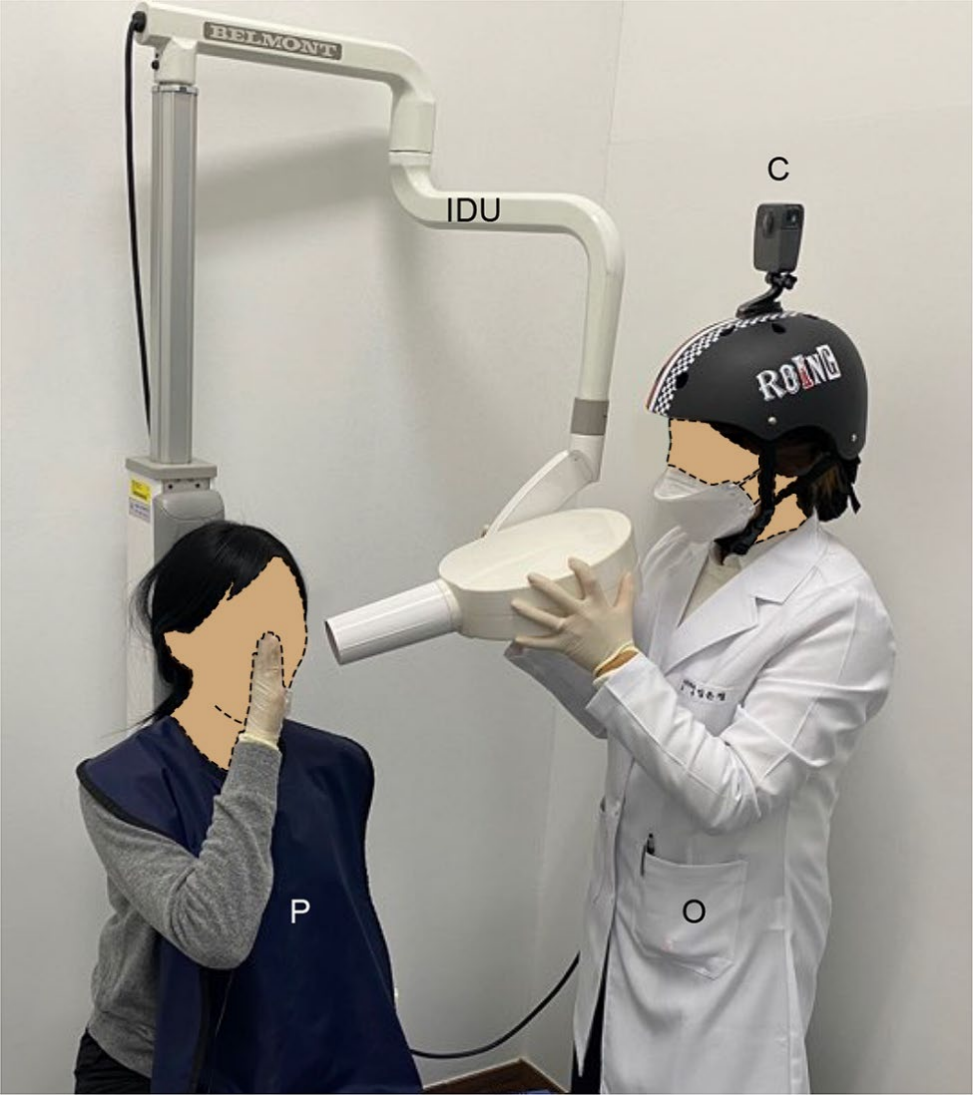
A 360° video recording. Dental radiography using the bisecting angle technique from the operator’s point of view using a 360° camera. The operator controls the intraoral dental X-ray unit to explain the dental radiography process. C: 360° camera, IDU: Intraoral dental X-ray unit, O: Operator, P: patient

This randomized controlled trial compared the control and experimental groups in a dental radiology class. To provide a VR experience using pHMD on a smartphone, we produced a 360° video for DR that can be played on a mobile device. The operator (dental hygienist; 17 years of DR experience) who performed DR wore a helmet (Zoot roing helmet, Foshan Nanhai Sunshine Sports Goods Co., LTD, Guangdong, China) fixed with a 360° camera. The training process for re-enacting the bisecting angle technique from the operator’s point of view was recorded as a 360° video, which was recorded using a GoPro Fusion camera (GoPro, San Francisco, the USA) (see Fig. 1). In addition, the DR process was recorded as 2D images by the same operator and DR from the operator’s point of view was recorded using a smartphone (iPhone XS, Apple Inc., Cupertino, USA). The 360° video was edited using Premiere Pro (CC 2020, Adobe, San Jose, the USA) and After Effects (CC 2020, Adobe, San Jose, USA) software. The operator’s explanation of the DR method and precautions for the DR technique were superimposed on the 360° image and inserted as an animation effect. This editing process was also performed in a 2D recorded video. The 360° videos were subsequently edited to be the same length (i.e., 40 min and 17 s each) and converted into a 360° VR format to enable use with the pHMD. Both the 2D (recorded using a smartphone) and 360° VR (recorded using a dedicated 360° camera) videos were captured simultaneously to document the operator’s DR process. The 2D and 360° videos were uploaded to YouTube (https://www.youtube.com). In particular, to provide learning through the VR experience of DR in an online environment, we configured the VR medium to enable the VR experience using the YouTube application and pHMD of smart phone.

Participants were randomly classified into the control and experimental groups. The control group learned using a 2D video. The experimental group learned through 360° videos based on the VR using pHMD (Google Cardboard, Google LLC, California, the USA). Using Zoom video communications (Zoom, San Jose, the USA; https://zoom.us), the YouTube link where the recorded 2D and 360° videos were uploaded was shared with the participants in the control and experimental groups, respectively. To ensure the integrity of the pHMD, a two-tier packaging approach was employed before distribution to the students in the experimental group. Explicit instructions cautioning against opening the outer box before the commencement of the experiment were provided. Additionally, security measures were implemented by applying a circular wax seal within the inner box, taking advantage of the characteristic residue left when the box was opened. Box integrity was verified remotely via video communication (using Zoom), following which the participants were granted access to the VR functionality and provided with a YouTube link.

**Fig. 2:**
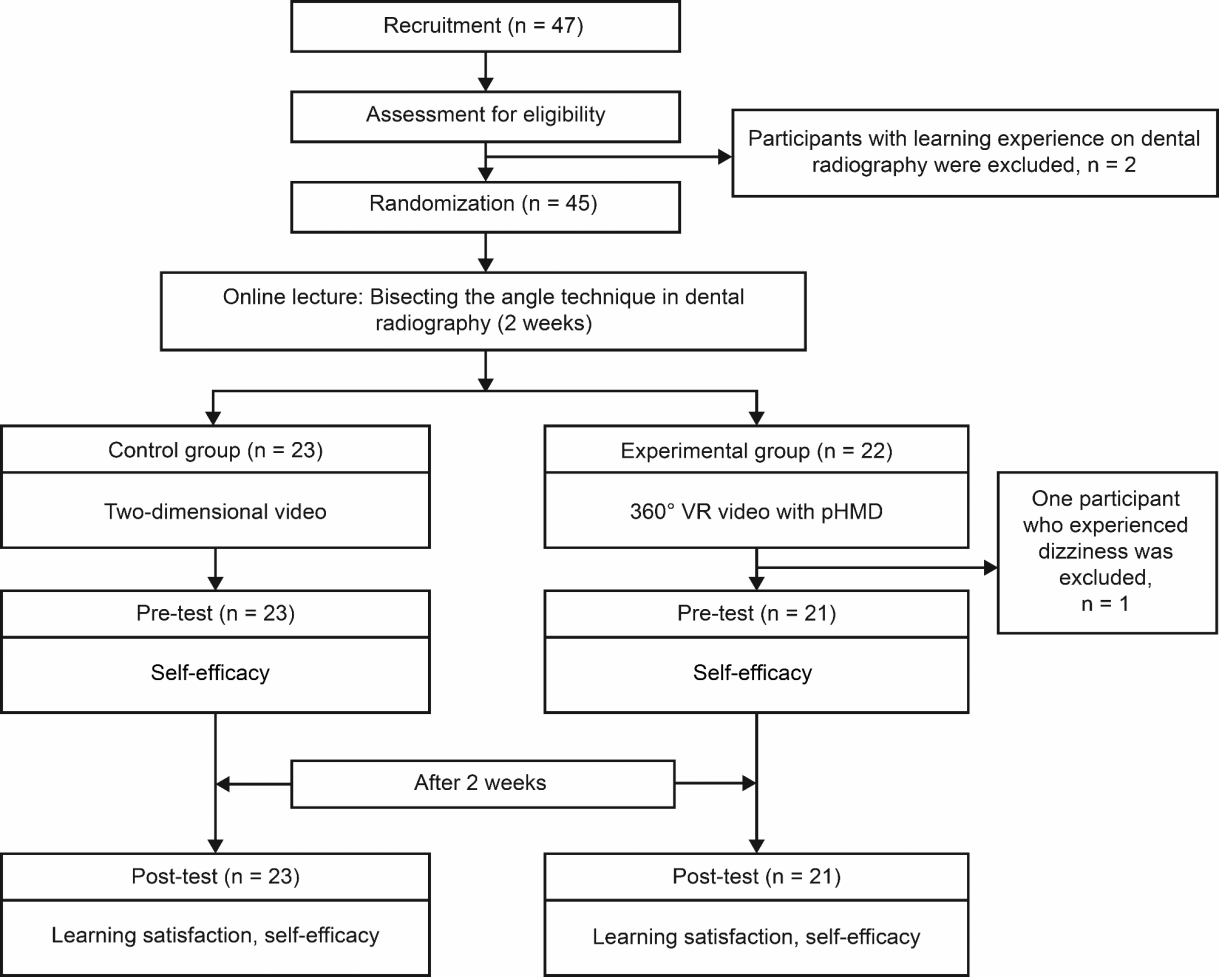
Participant recruitment and survey process. pHMD: Prefabricated head-mounted display

The process of installing the YouTube application and using the VR function were explained to the participants in the experimental group. Hence, it can be used as an HMD by attaching a smartphone. Theoretical concepts of the bisecting angle technique were explained by instructors in 3-h online lectures viewed by participants in regular dental radiology classes. The online lecture video provides a theoretical explanation of the bisecting angle technique by the instructor. After regular dental radiology classes, all the participants additionally studied 1 h/week using 2D and 360° VR videos. These 2D and 360° VR videos are recorded re-enactments of the operator and patient’s bisecting angle technique. The control group learned through a 2D video, while the experimental group used the 360° VR video. In both groups, learning satisfaction was assessed once after the participants had used their respective mediums while self-efficacy was measured twice before and after exposure to each medium (see Fig. 2). There are two hypotheses: (1) H0: There is no difference in learning satisfaction experienced by the participants using different DR learning tools; H1: There are differences in learning satisfaction experienced by the participants using different DR learning tools. (2) H0: There is no difference in self-efficacy experienced by the participants using different learning tools of DR. H1: There is a difference in self-efficacy experienced by the participants using different learning tools of DR. H0 represents the null hypothesis, while H1 represents the alternative hypothesis. This study was approved by the Institutional Review Board of Namseoul University (202102-003).

### Recruitment of participants

A recruitment notice was posted on the online bulletin board for students taking dental radiology in the 2nd year of the Department of Dental Hygiene at Samhyook University in 2021. Students who voluntarily wanted to participate were recruited for 1 week. The notification specifies that the participants’ academic grade was unaffected. The current study provided no financial benefit for participation. In total, 47 voluntary participants were recruited. An online informed consent was obtained from all the participants. Two participants with experience learning DR were excluded. Then, 45 participants were randomly classified into two groups, i.e., control (n = 23) and experimental (n = 22) groups (see Fig. 2), using the random numbers function of Excel software (Microsoft Excel 2016, Redmond, the USA) by the instructor in charge of dental radiology. In the experimental group, one participant who felt dizzy while using the 360° VR stopped the experiment. Therefore, the final number of participants in the experimental group was 21. The YouTube links for the 2D and 360° VR videos were only released to each group for 2 weeks. To ensure accurate analysis of the experiment, it was requested that participants in the experimental group refrained from lending the pHMD to the control group or sharing the YouTube link. After the survey ended (posttest), all links were made private. After the experiment was completed, all imaging sources and pHMD were provided to all students taking the dental radiology class.

### Survey tool for comparison between groups

Wang et al.’s [32] survey items regarding learning satisfaction comprise a total of six questions. Consequently, one survey item (i.e., the course format is helping me to prepare for my exam better and receive a higher score) was excluded, resulting in a total of five questions (see Table 1), and the Cronbach’s alpha value was 0.908. The self-efficacy questionnaire was designed using eight questionnaires previously developed by Chen et al., (see Table 1) [33], and the Cronbach’s alpha value was 0.912. Quantitative evaluations through test questions were not performed in the current study.

**Table 1:** Survey questions on learning satisfaction and self-efficacy

Variable	Questionnaire items
Learning satisfaction	L1. The instruction approach currently used contributes to my learning course content
	L2. I feel relaxed and comfortable in this approach
	L3. The course format is helping me to prepare my future professional life better
	L4. I spend less time on learning this course
	L5. The course format is helping me to improve my communication skills
Self-efficacy	S1. I will be able to achieve most of the goals that I have set for myself
	S2. When facing difficult tasks, I am certain that I will accomplish them
	S3. In general, I think that I can obtain outcomes that are important to me
	S4. I believe I can succeed at any endeavor to which I set my mind
	S5. I will be able to successfully overcome many challenges
	S6. I am confident that I can perform effectively on many different tasks
	S7. Compared to other people, I can do most tasks very well
	S8. Even when things are tough, I can perform quite well

L: Learning satisfaction; S: Self-efficacy

### Statistical analysis

The self-administered questionnaire was designed to enable the assessment of agreement levels using a 5-point Likert scale. Learning satisfaction was assessed once after exposure to the relevant medium, while self-efficacy was measured twice before and after exposure to ensure group homogeneity. The same questionnaire was used in both groups, and intergroup comparison of learning satisfaction and self-efficacy after exposure was performed.

The Naver form (Naver, Seongnam, Republic of Korea, available at: https://office.naver.com), a self-reported online research platform, was used. The data collected were analyzed using Statistical Package for the Social Sciences software (SPSS version 23.0, IBM Co., Armonk, NY, the USA). All statistical tests were performed with a 95% confidence interval (with the significance level set as 0.05). The normality test was performed using Shapiro–Wilk test (p < 0.05), and data were evaluated using the nonparametric statistical analysis method. The Mann–Whitney U test was used to evaluate differences in pretest self-efficacy (i.e., the participant’s subjective state before intervention) and posttest self-efficacy and learning satisfaction (i.e., the participant’s subjective state after intervention) between the control and experimental groups. There were no missing data during the experiment.

## Results

**Fig. 3:**
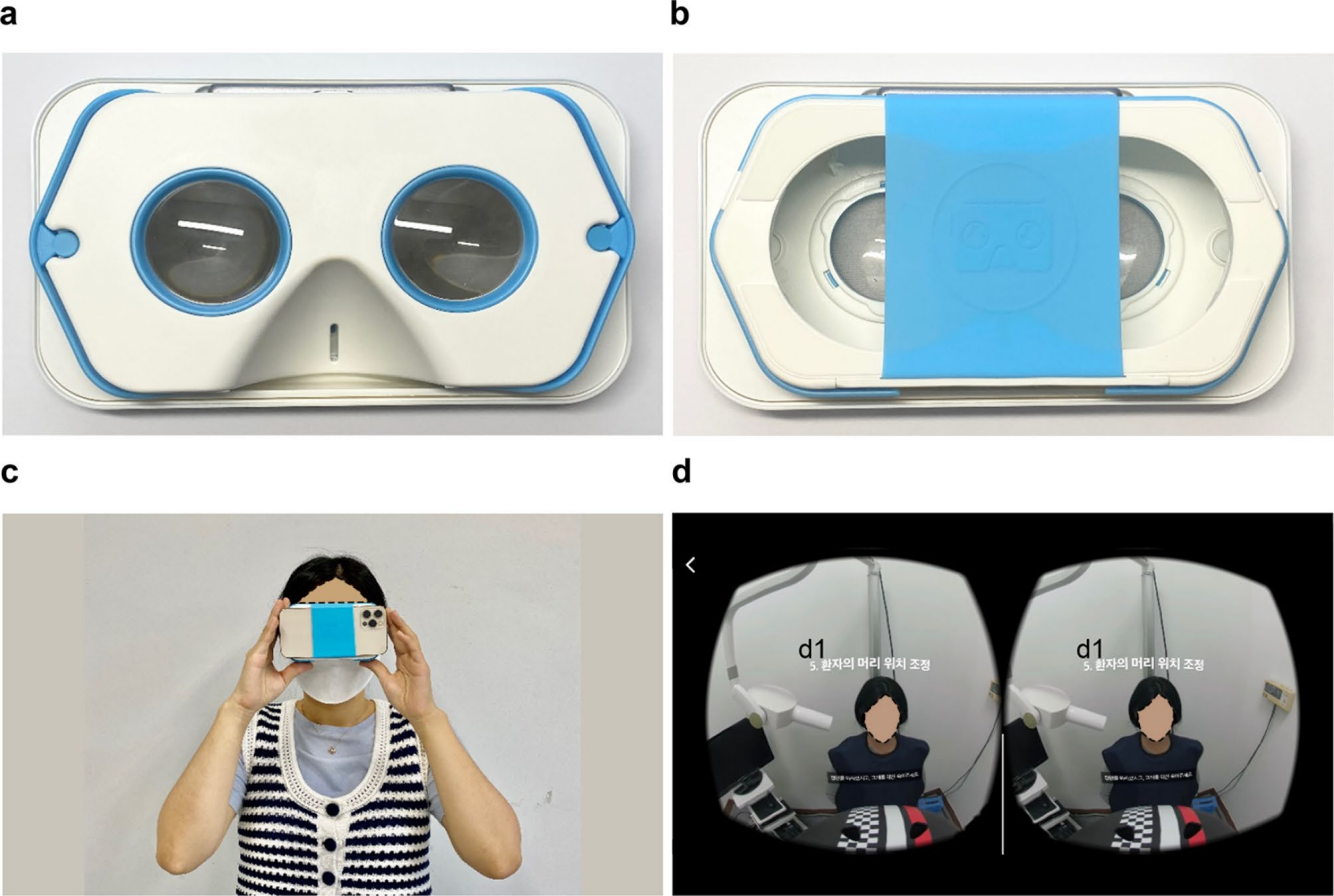
Dental radiography learning tool based on virtual reality. (**a**) Anterior aspect of prefabricated head-mounted display (pHMD). (**b**) Posterior aspect of pHMD. (**c**) The learner attaches a smartphone to pHMD and uses it as a head-mounted display. (**d**): This photo shows that a 360° recorded video is played by dividing the same video into the left and right eyes on a smartphone attached to pHMD. d1: The sentence “d1” stands for “guide and adjust the patient’s head appropriately for dental radiography” in Korean

**Fig. 4:**
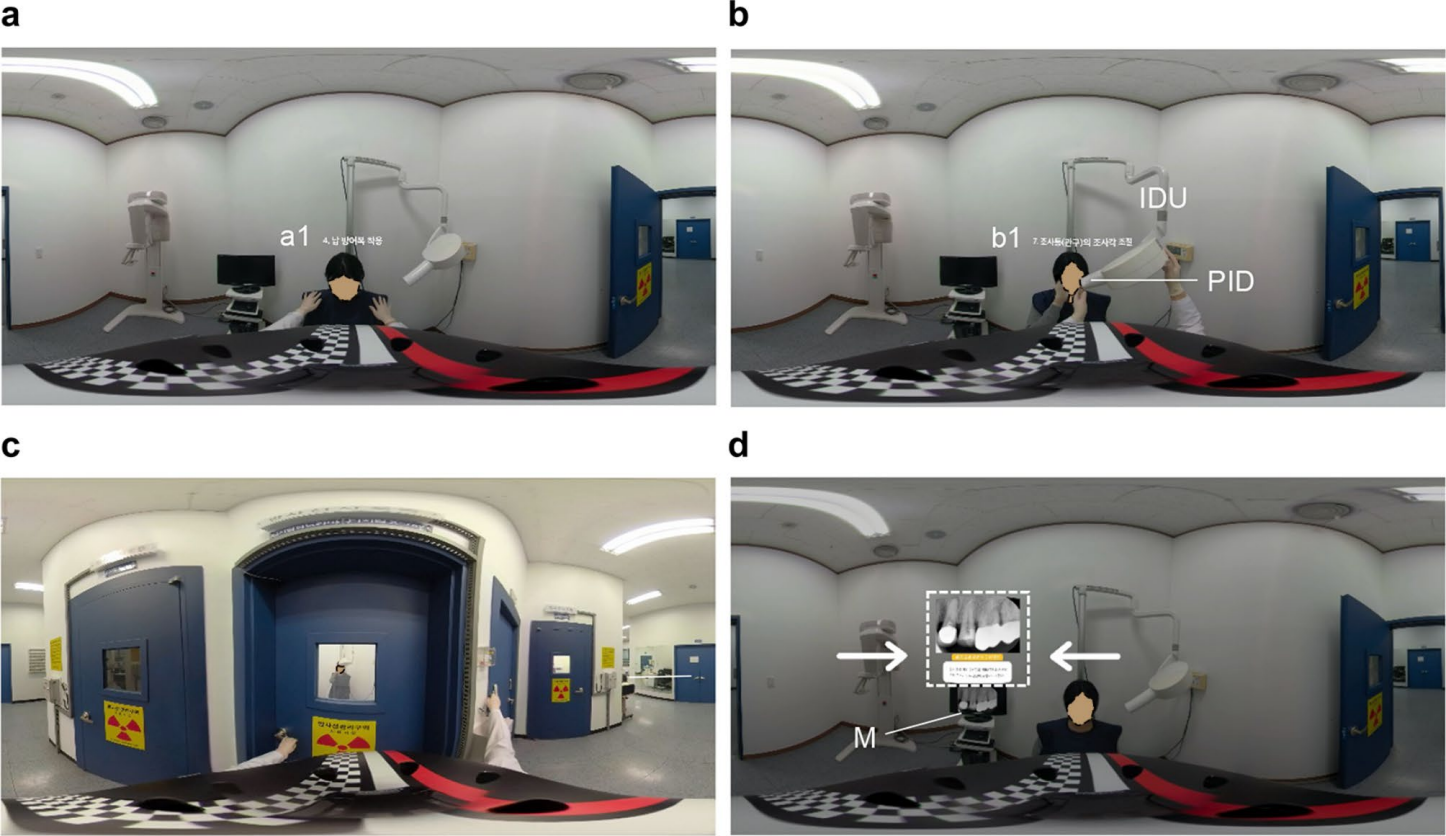
360° video according to the sequential dental radiography process. (**a**) Preparatory steps to help patient put on lead apron. (**b**): Step of manipulating the position indicating the device (PID) of the intraoral dental X-ray unit (IDU). (**c**) Steps taken outside the dental X-ray room. (**d**): Guide for checking on dental radiograph (white dotted box) and dental radiograph results on the monitor (M). a1: The sentence “a1” stands for “Put on lead apron” in Korean. b1: The sentence “b1” stands for “Manipulation of PID” in Korean

By clicking the VR icon in the YouTube application, DR image recorded in 360° on the smartphone was divided into left and right. The participants could experience VR after playing the video by attaching the smartphone to the pHMD. Figure 3 shows the pHMD being used by the participant, how to use it, and how a 360° video is played through the pHMD. Figure 4 presents a visual depiction of the sequential steps involved in DR that learners can reference via pHMD. Figure 4a shows the operator donning a lead protective suit on a patient before operating a dental X-ray machine; Fig. 4b depicts the procedural steps involved in operating a dental X-ray machine; Fig. 4c shows the operator pressing the imaging button from outside the dental X-ray room; and Fig. 4d shows the superimposition of oral radiographs after performing DR.

The mean age of all participants was 22.2 years; 95.5% (n = 42) were women and 4.5% (n = 2) were men. The mean age of the control group was 22.1 years, and 95.7% (n = 22) were women and 4.3% (n = 1) were men. The mean age of the experimental group was 22.3 years, and 95.2% (n = 20) were women and 4.8% (n = 1) were men. Both groups were second-year dental hygiene students with no prior experience taking a dental radiology class.

Comparing pretest self-efficacy between groups using Mann–Whitney U test revealed median scores of 2.56 and 2.67 in the experimental (n = 21) and control (n = 23) groups, respectively. However, this difference was not statistically significant (U = 219.00, z = − 0.530, p = 0.596), suggesting no discernible distinction between the experimental and control groups in terms of self-efficacy before intervention. The posttest learning satisfaction scores were significantly higher (U = 38.50, z = − 4.786, p < 0.001) in the experimental group (median = 4.00, n = 21) compared with the control group (median = 2.60, n = 23; Table 2), as were the posttest self-efficacy scores (experimental group: median = 3.78, n = 21; control group: median = 2.67, n = 23; U = 62.00, z = − 4.232, p < 0.001; Table 2).

**Table 2:** Comparison of posttest results for learning satisfaction and self-efficacy by Mann–Whitney U test between groups

Variable	Group	Descriptive statistics			Mann–Whitney U test						
		n	M$\pm$SD	Median		Mean rank	Sum of ranks	U	z	*p*	
Learning satisfaction	C	23	2.53$\pm$0.78	2.60		13.67	314.50	38.50	−4.786	0.000*	
	E	21	3.98$\pm$0.73	4.00		32.17	675.50				
Self-efficacy	C	23	2.75$\pm$0.78	2.67		14.70	338.00	62.00	−4.232	0.000*	
	E	21	3.77$\pm$0.47	3.78		31.50	652.00				

*p < 0.001

C: Control group, E: Experimental group, M: Mean, n: Number of participant, SD: Standard deviation

## Discussion

Self-learning tools aimed at facilitating independent learning among students are essential in the field of dental education [34, 35], and 360° VR technologies can effectively foster self-directed learning by providing easily accessible immersive experiences of medical scenarios [36]. Content generated using 360° video technology typically replicate real-life situations, thus creating a highly realistic immersive learning environment [17, 37]. The impact of these videos on learning outcomes can be elucidated using the framework of situated learning theory [38] wherein students derive knowledge from authentic learning activities following active engagement in the learning experience [39]. Situated learning occurs within the same context where knowledge is acquired and applied. Users of 360° video technology can immerse themselves in VR experiences by viewing images captured using a 360° camera through HMDs such as Oculus Rift or Google Cardboard [40]. In particular, learning facilitated by pHMDs (e.g., Google Cardboard) and 360° videos are accessible via smartphones, and previous studies using these educational mediums have reported high levels of course material comprehension among users and also provided recommendations regarding media usage [41, 42]. Therefore, 360° videos and pHMDs offer several advantages when used for the purpose of education in a nonface-to-face online environment.

Furthermore, among advanced VR technologies, the use of mixed-reality technology using Microsoft’s HoloLens provides an augmented and virtual experience in dental education and is highly effective [43, 44]; hence, it is a good alternative in DR learning. Nevertheless, educational and training institutions face significant financial challenges with regard to acquiring both equipment and content [45, 46], making the provision of these resources to students challenging. This is also applicable to HMDs such as Oculus Rift. Therefore, there is a need for a new VR medium in DR education and learning. Advantages of VR are not subject to environmental restrictions and it allows a 3D approach, thereby enabling self-directed learning [47, 48]. In particular, 360° videos are easily produced and pHMDs are cost effective (low cost) and enable the comprehension of visual and spatial dimensions, thus enhancing cognitive skills [49]. Hence, 360° VR with pHMD can provide a VR learning medium to many students, and as it uses a smart phone, it is possible to use it flexibly without environmental restrictions. Herein, a 360° VR with pHMD that can be used online in a nonface-to-face educational environment in DR learning was developed, and learning satisfaction and self-efficacy of the learners were evaluated.

In the evaluation of the learning satisfaction and self-efficacy in the experimental group, the use of VR learning media using pHMD revealed a statistically significant difference. The experimental group using 360° VR with pHMD showed significantly higher learning satisfaction and self-efficacy than the control group (see Table 2). Learning satisfaction refers to the degree of influence on a learner’s learning motivation or academic achievement. Further, it is correlated with the teaching method, educational content, learning environment, and learning convenience, and is a factor that improves learning motivation [50, 51]. Self-efficacy is a judgment of an individual’s ability to organize and execute behaviors necessary for the learner to perform a task. It affects efforts used to select and perform new tasks and academic achievement [33, 52, 53]. However, this does not suggest that utilizing 360° learning media for DR education directly influences academic performance, but instead emphasizes the usability and potential efficacy of this medium in this context. Further research is necessary to elucidate the relationship between self-efficacy and academic achievement in DR. Herein, compared with the conventional 2D videos, 360° VR is associated with a higher learning satisfaction and self-efficacy in DR learning.

The experimental group using 360° VR with pHMD exhibited greater satisfaction with the learning tool than the control group that used existing 2D videos (Table 2). The survey questions related to learning satisfaction (L1–5 in Table 1) showed that 360° VR with pHMD significantly contributed toward the learning process, was easily accessible, reduced course duration, and enhanced communication skills. Furthermore, the course format used for this learning medium was considered beneficial with regards to preparing for future professional endeavors. The evaluation of self-efficacy revealed higher scores in the experimental group using 360° VR with pHMD compared with the control group using 2D videos (Table 2). Self-efficacy was assessed by analyzing participant responses to certain statements (S1–S8 in Table 1) that gauged an individual’s confidence in their ability to attain personal objectives, tackle challenging tasks, achieve desired outcomes, and overcome obstacles. Participants expressed confidence in their ability to perform various tasks proficiently, compare their skills with others, and maintain performance even in challenging situations. These findings suggest that learning media that utilize 360° VR with pHMDs are a suitable alternative for existing 2D videos in DR education courses.

A 360° VR with pHMD can be produced easily and rapidly from the instructor’s perspective in DR education. However, it has is a limitation. That is, the learner does not directly operate in an intraoral dental X-ray unit. Oral radiographic images that contain errors cannot precisely validate the patient’s condition, thus affecting accurate diagnosis [54]. Obtaining intraoral radiographic images that accurately depict the patient’s oral condition is largely dependent on the skills of the DR operator. However, learning media using 360° videos with pHMDs are limited by their inability to provide a virtual learning experience where the student can directly operate a dental X-ray unit. Errors in the DR technique may arise from inaccurate vertical or horizontal angles caused by the misalignment of the position indicator of the dental X-ray device or improper placement of the image receptor, as shown by previous studies examining various imaging methods [55, 56]. Hence, further research on the development of an interactive DR simulator that utilizes existing HMDs and is based on 3D objects is necessary. The quantitative assessment of the educational effectiveness of learning mediums using 360° videos with pHMDs was not performed in the current study owing to the challenges associated with controlling variables such as the use of other learning resources (e.g., Internet, books) and total study duration within a nonface-to-face learning environment. Tak et al. [18], previously used 360° videos with pHMDs as a prelearning tool to enhance periodontal instrumental skills in a face-to-face environment and conducted quantitative evaluation of the students’ academic performance. In this context, further research is warranted to assess the effectiveness of 360° VR learning media for DR education.

Statistically significant differences in learning satisfaction and self-efficacy were observed between students using 360° videos with pHMD and those using traditional 2D videos in a nonface-to-face environment. However, it is important to note that these differences do not necessarily imply an educational effect. These findings suggest that 360° VR with pHMDs may be considered a suitable alternative for nonface-to-face DR education in the absence of learning media other than 2D videos.

## Conclusions

The current study utilized 2D and 360° VR videos to develop learning mediums that enabled virtual DR education in a nonface-to-face learning environments. The participants underwent VR learning using their smartphones and pHMDs, and their levels of learning satisfaction and self-efficacy were assessed using surveys. There was a statistically significant difference between the control group, which used 2D video, and the experimental group, which used 360° VR with pHMD, with the experimental group rating these two factors highly. The 360° VR with pHMD could be easily installed on the students’ smartphones and used without any environmental restrictions. The findings revealed that this method was associated with greater learning satisfaction and self-efficacy than DR learning mediums using 2D videos, suggesting that it may be considered a suitable alternative for education in nonface-to-face online environments. Quantitative evaluation of academic achievement (e.g., through exams) was not performed as it was realistically impossible to prevent the students from consuming any media other than the ones provided in a nonface-to-face environment. Additional research is needed on the educational effectiveness of differences between media in a face-to-face environment. Additionally, other methods of utilizing 360° VR learning media as preliminary training materials before actual DR practice should be explored. Finally, research aimed at the development of a VR simulator that allows learners to interact with the DR process directly is also necessary.

## Data Availability

The datasets utilized in this study can be obtained by contacting the corresponding author through a reasonable request.

## List of abbreviations

2D: Two-dimensional
3D: Three-dimensional
DR: Dental radiography
HMD: Head-mounted display
L: learning satisfaction
pHMD: Prefabricated head-mounted display
S: self-efficacy
VR: Virtual reality
